# To Our Editorial Brethren—Postal Annoyances

**Published:** 1877-08-20

**Authors:** 


To Our Editorial Brethren.—Postal Annoyances.—We take it for granted that all of our cotemporaries in journalism have had trouble with the Post-office Department in regard to postage on institched matter. If notice had been given that, at a set period ahead, these inserts would be required to pay increased rates of postage, then we could have adapted ourselves to the requirement without loss or inconvenience by closing out our contracts with advertising patrons, or otherwise arranging with them. As it is, the construction put upon the law, and its hasty enforcement, has caused serious loss to many journals. We now propose to our editorial brethren to unite with us in an effort through our journals, and by operating on our representatives in Congress, to procure the passage of a law simplifying our postal regulations and removing all petty restrictions and technical exactions. A law *liberal, uniform, cheap* in the matter of postage, and free as possible from doubtful construction. We would not be understood as censuring the present incumbents, as they must enforce the law as they find it; but the law itself should be modified in many particulars. Especially let us, as journalists, insist upon having as easy rates of postage as the newspapers, which pay only two cents, while we pay three cents per pound. An able gentleman, experienced in postal matters, has promised us to draft a better law, and a representative in Congress, from Georgia, will introduce the bill and defend it. We respectfully urge upon editorial brethren to use their influence in its behalf.

